# Tonsil Stromal-Cell Lines Expressing FDC-Like Properties: Isolation, Characterization, and Interaction with B Lymphocytes

**DOI:** 10.1155/1998/81637

**Published:** 1998

**Authors:** Grzegorz Skibinski, Anna Skibinska, Martine Deckers, Keith James

**Affiliations:** Lister Research Laboratories Department of Surgery University of Edinburgh Royal Infirmary, Lauriston Place, Edinburgh Scotland, EH3 9YW UK

**Keywords:** FDC-like cell lines, FDC/B lymphocyte interactions, human tonsil

## Abstract

The microenvironment of secondary lymphoid organs consists of two major populations of cells, the lymphoid cells and a population of stromal cells that contribute to both tissue architecture and function. Interactions of both populations are essential for the development and control of humoral immune responses. In this study, stromal-cell preparations were obtained by a multistage process. This involved culturing 300-400-*μ*m slices of human tonsil for 6-8 days at 25^°^C, trypsin digestion of the residual explant, followed by CD45-positive-cell depletion using magnetic beads, and a final period of culture for 4 days to remove remaining nonadherent cells. Phenotypifig with a panel of monoclonal antibodies revealed that the cells express HLA-DR, CD54 (ICAM-1), CD44, but no CD45 nor a range of other markers for epithelial and endothelial cells. Immunoassays of supernatants from stromal cells revealed that IL-6 was produced constitutively, and its production was increased by treatment with TNF-*α* and IFN-*γ*. In contrast IL-1, IL-2, IL-4, IL-7, IL-8, IL-10, IL-12, TNF-*α*, and IFN*γ* were not produced. Functional tests showed that these cells express follicular dendritic cell-like properties. Coculturing of tonsilar B cells with stromal cells resulted in enhanced proliferation and also led to increased production of immunoglobulins and IL-6, suggesting crucial signaling between these populations.


*Developmental Immunology*, 1998, Vol. 6, pp. 273–284
Reprints available directly from the publisher
Photocopying permitted by license only

© 1998 OPA (Overseas Publishers Association)
N.V. Published by license under the
Harwood Academic Publishers imprint,
part of the Gordon and Breach Publishing Group
Printed in Malaysia

### Tonsil Stromal-Cell Lines Expressing FDC-Like Properties: Isolation, Characterization, and Interaction with B Lymphocytes

GRZEGORZ SKIBINSKI*, ANNA SKIBINSKA, MARTINE DECKERS and KEITH JAMES

*Lister Research Laboratories, Department of Surgery, University of Edinburgh, Royal Infirmary, Lauriston Place, Edinburgh EH3 9YW, Scotland, UK*

*(Received 5 August 1996; In final form 4 May 1997; Accepted 14 August 1997)*

The microenvironment of secondary lymphoid organs consists of two major populations of cells, the lymphoid cells and a population of stromal cells that contribute to both tissue architecture and function. Interactions of both populations are essential for the development and control of humoral immune responses. In this study, stromal-cell preparations were obtained by a multistage process. This involved culturing 300-400-$\mu$m slices of human tonsil for 6-8 days at 25°C, trypsin digestion of the residual explant, followed by CD45-positive-cell depletion using magnetic beads, and a final period of culture for 4 days to remove remaining nonadherent cells. Phenotyping with a panel of monoclonal antibodies revealed that the cells express HLA-DR, CD54 (ICAM-1), CD44, but no CD45 nor a range of other markers for epithelial and endothelial cells. Immunoassays of supernatants from stromal cells revealed that IL-6 was produced constitutively, and its production was increased by treatment with TNF-$\alpha$ and IFN-$\gamma$. In contrast IL-1, IL-2, IL-4, IL-7, IL-8, IL-10, IL-12, TNF-$\alpha$, and IFN$\gamma$ were not produced. Functional tests showed that these cells express follicular dendritic cell-like properties. Coculturing of tonsilar B cells with stromal cells resulted in enhanced proliferation and also led to increased production of immunoglobulins and IL-6, suggesting crucial signaling between these populations.

*Keywords:* FDC-like cell lines, FDC/B lymphocyte interactions, human tonsil

### INTRODUCTION

During antigen-specific T-cell-dependent immune responses in secondary lymphoid tissues, antigen-specific naive B cells undergo a series of events including activation, expansion, somatic mutations, isotype switch, selection, and differentiation into either antibody-secreting or memory cells (Kroese et al., 1990; Möller, 1992). This maturation process is highly controlled and depends on numerous interactions with interdigidating dendritic cells, T cells, and stromal cells, including highly specialized follicular dendritic cells (Kosco and Gray, 1992). The crosstalk between B-cell subsets and their microenvironment is controlled by membrane/ligand receptor interactions (MacLennan, 1994) and signals from

*Corresponding author.

the cytokine network (Banchereau and Rousset, 1992).

Most of the studies on follicular dendritic cells *in vitro* have been hampered by the fact that it is very difficult to isolate pure FDC with reasonable yields. Tsunoda et al. (1990) first reported isolation and maintenance of human FDC in long-term culture; these cells, however, did not grow *in vitro*. Lindhout et al. (1994) obtained EBV-transformed human FDC lines, but their long doubling time makes their cultivation *in vitro* difficult. Clark et al. (1995) and Kim et al. (1995) described cell lines with FDC-like properties that grew *in vitro* without exogenous growth factors.

In this paper, we followed the idea used by Jenkinson et al. (1992) in the mouse and by Gady et al. (1993) in human systems to obtain thymic stromal elements. We cut tonsil tissue into 300-400-$\mu$m thick slices and then cultured them at 25°C in order to deplete them of CD45-positive cells. CD45-negative cells obtained in this way were cultured *in vitro*. Stromal elements thus obtained were enzyme-digested and expanded *in vitro*. In this study, we show that the stromal-cell lines (SCL) obtained by this procedure exhibit FDC-like properties. Furthermore, we have characterized their phenotype, cytokine secretion, and their interaction with B lymphocytes.

### RESULTS

### Isolation of Human Tonsil Stromal Cells

Tonsils were cut into 300-400-$\mu$m thick slices and incubated in RPMI containing 1% FBS for 6-8 days at 25°C with gentle rocking. During this time, most of the lymphocytes were shed into the medium. At the end of the culture period, the tissue slices were washed and digested with 0.25% trypsin. The cells obtained still contained a variable proportion of CD45-positive cells (5-20%, depending on the experiment); it was therefore further subjected to a depletion step with the aid of magnetic beads. The resulting cell population contained less then 2% of CD45-positive cells and was seeded into culture flasks. After 24 hr incubation, large adherent cells appeared. During the first 3-4 days of culture, the few remaining lymphoid cells degenerated. The remaining cells were adherent and uniform, grew in the absence of exogenous cytokines, and exhibited characteristic fibroblastoid-like morphology (Fig. 1). Confluent cells were split using 0.25% trypsin to detach them from plastic. The results presented here were obtained from tonsils of seven different donors. The cells were passaged up to five times.

### Phenotypic Characterization of Isolated Stromal Cells

Phenotypic analysis was performed by flow cytometry on cells that were enzymatically detached from plastic, incubated overnight on a rocker platform, and stained with monoclonal antibodies listed in Table I. Initially, DRC-1, CD21, CD23, ICAM-1, VCAM, and HLA-DR were detected. Following a few days in culture, cells ceased to express DRC-1, a finding reported by other authors (Lindhout et al., 1994; Clark et al., 1995; Kim et al., 1995). Expression of CD21 and CD23 was also eventually lost. After about 2 weeks of culture, the cells expressed ICAM-1, CD44, and low levels of CD40 and HLA-DR. A low level of VCAM was also observed on two occasions (not shown). In the next set of experiments, the effect of activation with IFN-$\gamma$ and TNF-$\alpha$ was studied. Twenty-four hour culture with these cytokines upregulated ICAM-1, CD40, and HLA-DR expression (Figure 2).

### Cytokine Production in Cultures of Stromal-Cell Lines

SCL were cultured at 0.5 $\times$ $10^6$ cells/ml in the presence of TNF-$\alpha$, IFN-$\gamma$ or in the medium alone. After 3 days of culture, supernatants were collected and the cytokine concentration measured using a sandwich ELISA. In supernatants of unstimulated cultures, a substantial amount of IL-6 production was found. Stimulation of cells with TNF-$\alpha$ or IFN-$\gamma$ caused an increase in IL-6 secretion. IL-1$\beta$, IL-2, IL-4, IL-7, IL-10, IL-12, TNF-$\alpha$, and IFN-$\gamma$ were not

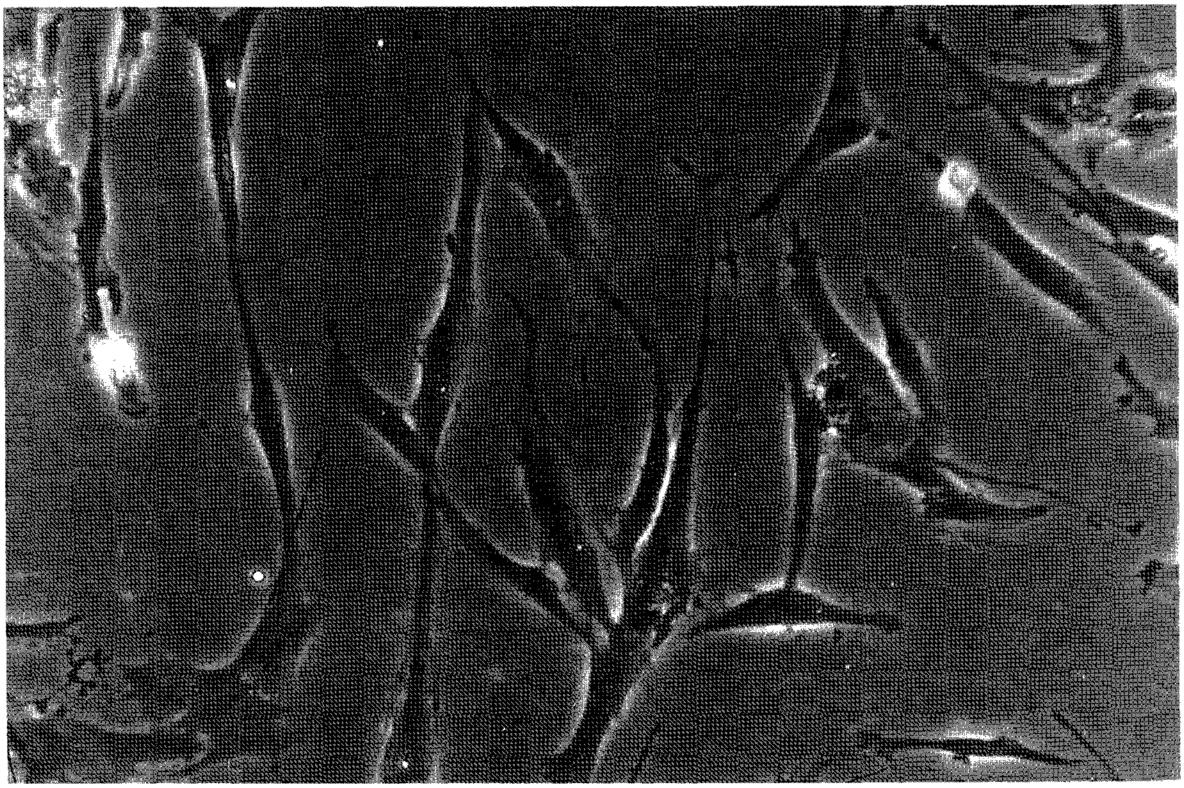

FIGURE 1 Cultured SCL. Magnification: 200×.

TABLE I Cell-Surface Markers Examined

| *T cells* | *B cells* | *Adhesion* |
|---|---|---|
| CD 2, *Leu-5b*[a] | CD 19, *HD37*[b] | CD 11a, *MHM24*[b] |
| CD 3, *UCHT1*[b] | CD 20, *B-Ly1*[b] | CD 11b, *OKM1*[f] |
| CD 4, *RPA-T4*[d] | CD 21, *BU35*[f] | CD 18, *MHM23*[b] |
| CD 8, *DK25*[b] | CD 23, *MHM6*[b] | CD 44, *BU52*[f] |
| *Myeloid* | CD 38, *Leu-17*[a] | CD 49d, *BU49*[f] |
| CD 14, *UCHM1*[e] | CD 39, *AC2*[f] | CD 54, *BBA4*[c] |
| CD 16, *BL-LGL/1*[e] | CD 40, *G28-5*[f] | CD 56, *NCAM-OB11*[e] |
| CD 35, *E11*[d] | *Nonlineage* | CD 58, *BRIC5*[d] |
| | CD 5, *OKT1*[f] | CD 106, *BBA5*[c] |
| | CD 10, *BE-3*[d] | |
| | CD 25, *ACT1*[b] | *Nonclustered* |
| | CD 45, *T29/33*[b] | anti-epithelial *E29*[b] |
| | CD 45, RO, *UCH-L1*[d] | anti-fibroblast *5B5*[b] |
| | CD 71, *DF1513*[d] | anti-FDC *DRC1*[b] |
| | HLA-DR, *CR3/43*[b] | |

The table contains names of monoclonal antibodies used together with names of corresponding clones (in *italics*) and their origin. Sources of monoclonal antibodies: [a]Beckton-Dickinson, Cowley, Oxford; [b]Dakopats, High Wycombe, Bucks; [c]British Biotechnology, Abingdon, Oxford; [d]Serotec, Kidlington, Oxford; [e]Sigma, Poole, Dorset; [f]obtained as a gift from J. Gordon and J. Pound, University of Birmingham, Birmingham, UK.

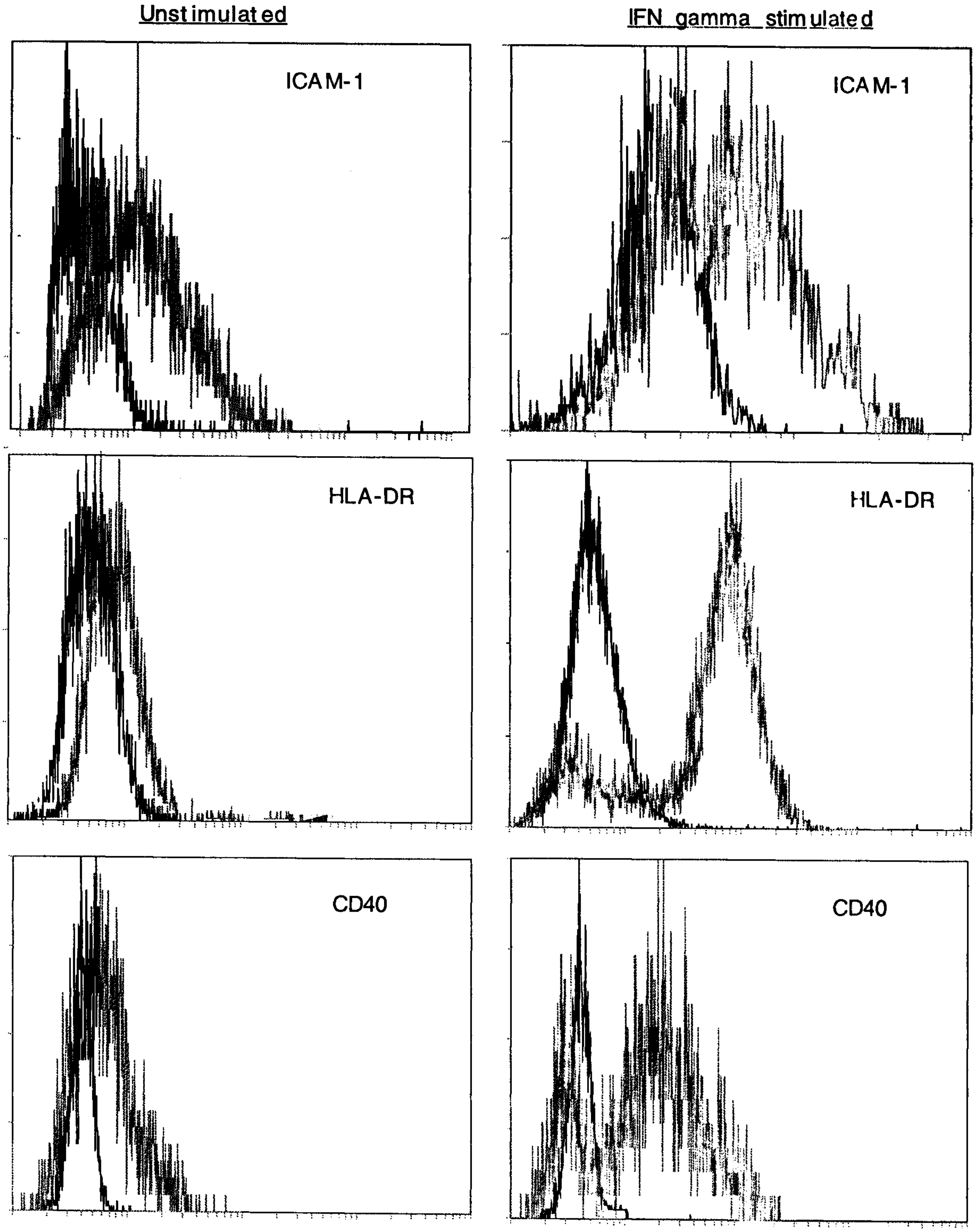

FIGURE 2 Expression of ICAM-1, CD40, and HLA-DR on unstimulated and stimulated (IFN-$\gamma$, 100 U/ml, 24 hr) SCL. Each histogram shows the negative control in black and expression of different markers in gray. The cell number is plotted on the $y$ axis; the log fluorescence intensity on the $x$ axis.

detected in culture supernatants of unstimulated cells (Table II).

### Effect of SCL-B-Cell Coculture on IL-6 Secretion

Combination of B lymphocytes with SCL led to a significant production of IL-6 within 3 days of coculture. B cells on their own did not produce detectable amounts of IL-6. In the same supernatants that had increased amounts of IL-6, no IL-2, IL-4, and IL-10 were detected (Figure 3).

TABLE II IL-6 Production by Cultures of Isolated Tonsilar Stromal Cells (pg/ml)

| Nonstimulated cultures | Cultures stimulated with TNFα | IFNγ |
|---|---|---|
| 400 ± 150 | 850 ± 154 | 910 ± 86 |

Results from three independent experiments (mean ± SD). Cells were cultured for 3 days in the presence of TNFα at 200 pg/ml or IFNγ at 50 U/ml.

### Effect of SCL on B-Cell Proliferation

The effects of SCL on proliferation of high-density B cells were investigated by measuring $^3$H-thymidine incorporation in mixed cultures. Cultures of B cells alone were run for comparison. SCL were irradiated (2000 rad) before experiment. This treatment reduced background $^3$H-thymidine incorporation without affecting B-cell proliferation. Figure 4 shows that B cells stimulated with SAC in the presence of SCL proliferated better than B cells cultured alone. Proliferation was increased even further by addition of recombinant IL-2 or IL-4. SCL supernatant enhanced

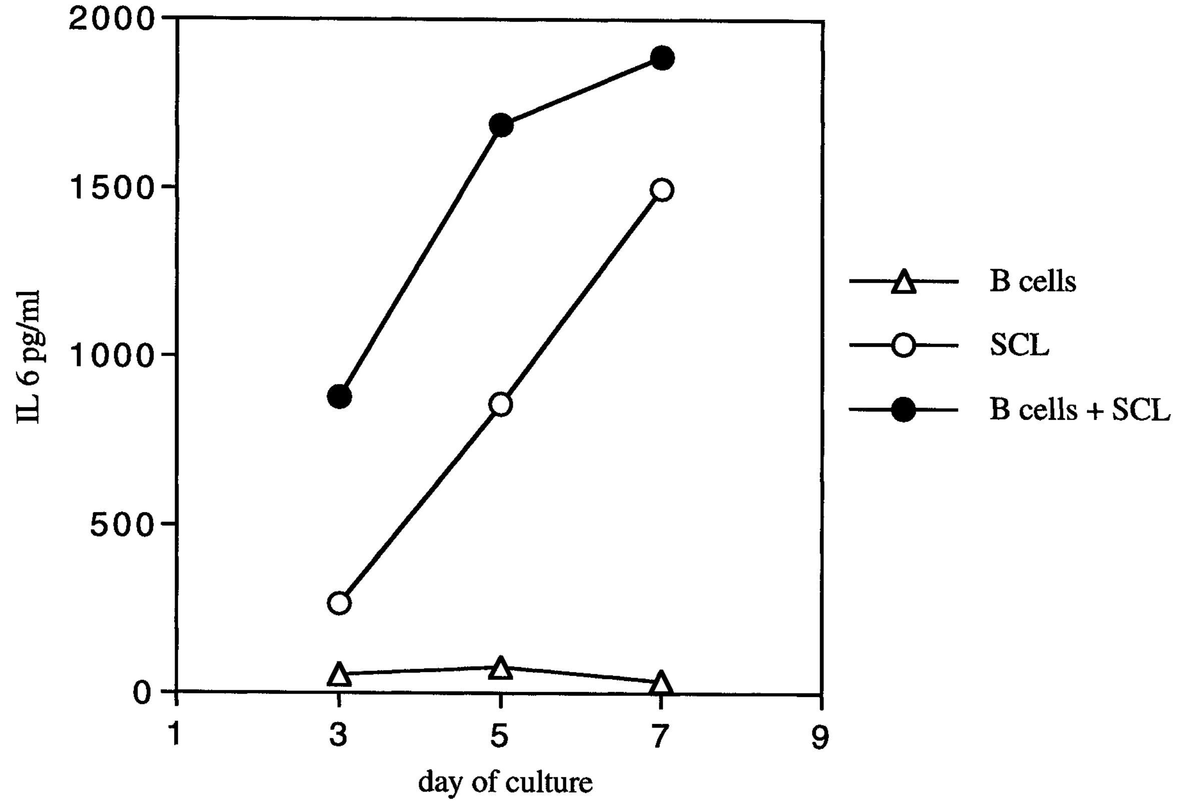

FIGURE 3 Coculture of SCL and B cells leads to increased secretion of IL-6. B cell (2.5 × $10^5$/well) and SCL ($10^4$/well) were cultured either alone or together. Culture medium was collected on the days indicated and assayed for IL-6 concentration by ELISA. IL-2, IL-4, and IL-10 were not detected in medium. Results are of one representative experiment out of five performed.

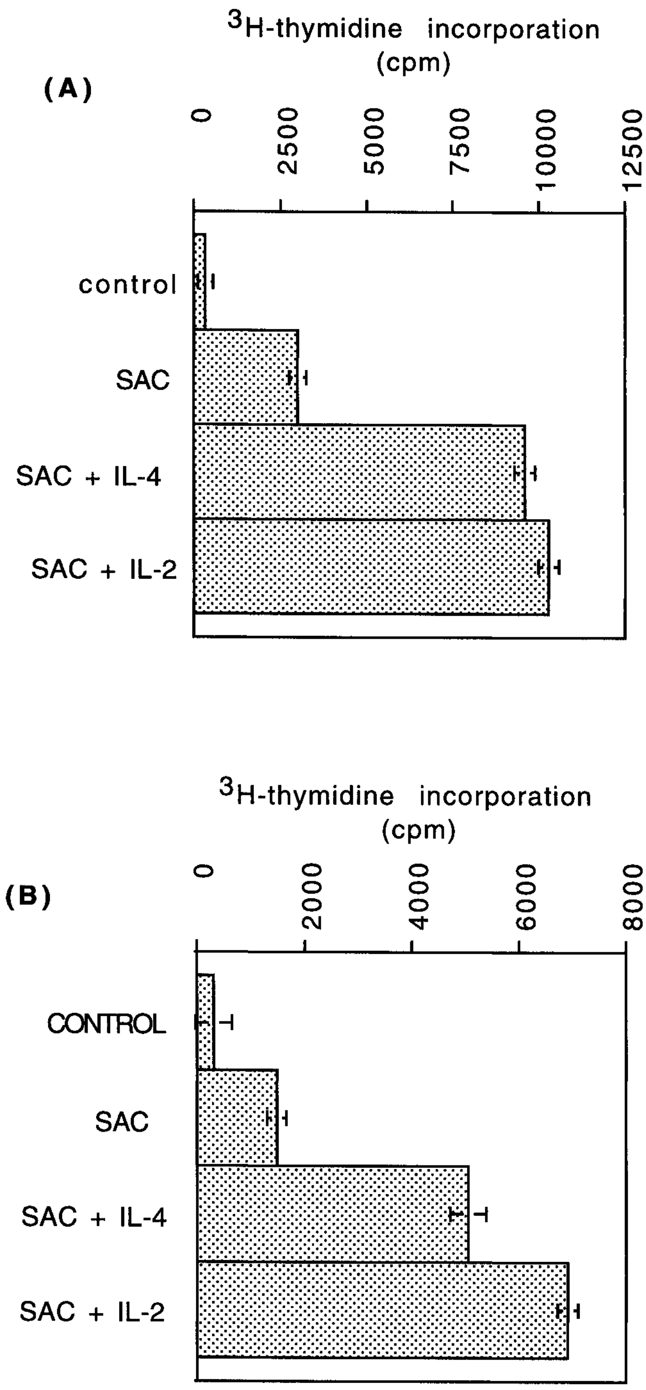

FIGURE 4 The effect of SCL on B-cell proliferation. (A) Irradiated SCL were seeded onto 96-well plates and incubated overnight at 37°C. B lymphocytes were then added together with SAC, IL-4 (50 U/ml), and IL-2 (100 U/ml). Cells were pulsed with $^3$H-thymidine for the last 8 hr of culture. (B) Proliferation of B cells in the presence of supernatants from stromal cell lines used in (A). Supernatant was used at optimal 1/3 dilution. Note the lower values of thymidine incorporation in the test with SCL. Figures are representative of three different experiments and are expressed as the mean cpm ± SD.

B-cell proliferation to a much lower extent suggesting the necessity for close cell-to-cell interaction for maximal effect (Figure 4B). There was no proliferation in SCL-B lymphocytes cocultures in the absence of a stimulant (data not shown).

### Effect of SCL on B-Cell Production of Immunoglobulin

One of the well-known properties of IL-6 is to promote B-cell maturation and immunoglobulin production. Because of this, we decided to measure the amount of IgG and IgM produced by high-density B cells in the presence or absence of stromal cells. Compared with B cells alone, B cells cultured together with SCL produced more IgG as well as IgM. Supernatant from SCL cultured alone contained only background levels of immunoglobulins. Addition of anti-IL-6-blocking antibodies to the system reduced the effect by 45% (Figure 5). Addition of recombinant IL-6 alone to B cells had only marginal effect on the amount of secreted Ig (data not shown).

### Comparison of Properties of SCL with Fibroblasts

We also compared the ability of human skin fibroblasts and SC lines to stimulate B-cell-induced proliferation and immunoglobulin secretion. Fibroblast clearly enhanced proliferation induced by SAC in the presence of exogenous IL-2 (100 U/ml). Also, spontaneous IgM secretion was increased in the presence of fibroblasts. Fibroblasts, however, were less effective in both tests in comparison to the stromal cell lines (Figure 6).

### DISCUSSION

In this paper, we demonstrate that CD45-negative adherent cell lines obtained from human palatine tonsils may interact and influence the activity of B lymphocytes. We decided to use the term SCL because it is difficult to pinpoint their origin. Although, they are not typical follicullar dendritic cells as far as surface phenotype is concerned, they do express several functional properties of native FDCs. Namely, they are large, nonphagocitic, with long cytoplasmic extensions. After isolation, they express markers characteristic for FDCs, namely, DRC1, CD21, and CD23. These cells also promote B-cell proliferation and enhance spontaneous secretion of immunoglobulins. Growth of these cell lines does not depend on exogenous growth factors. It is difficult to reach conclusions about FDC and the other cell types, especially, since they change phenotype depending on the microenvironment. Nevertheless, their functional analysis suggests that they may be derived from FDCs.

Contact between isolated stromal cells and B cells induces an increase in IL-6 production in culture, these being most likely from stromal cells. This fact suggests that IL-6 plays an important role in proliferation and/or differentiation of B cells present in our system. However, anti-IL6 antibody inhibited the increase in immunoglobulin production by 45%. Also, IL-6 could not replace stromal-cell induction of IgG and IgM production. This shows that IL-6 is not entirely responsible for increased immunoglobulin secretion and requires supplementation with other stimuli conceivably mediated through direct cell-to-cell contact. This is currently under investigation in our laboratory.

It has been suggested that what VLA-4 (CD49d) expressed by FDC *in situ* plays is crucial in FDC-B-cell interaction within the germinal center. Our lines, however, do not express CD49d and therefore this molecule is unlikely to play a role in processes described here. This topic requires further study.

Our lines also do not express Fc receptors or complement receptors; therefore, they are unlikely to be able to stimulate B cells in an antigen-specific fashion. However, it is known that germinal centers begin to form before trapping of antigen-antibody complexes (Kroese et al., 1986). Furthermore, during the early stages of germinal center formation, relatively few T cells are present, suggesting that FDC stimulate initial rapid B-cell proliferation and maturation without significant participation of T cells or immune complexes (MacLennan, 1994).

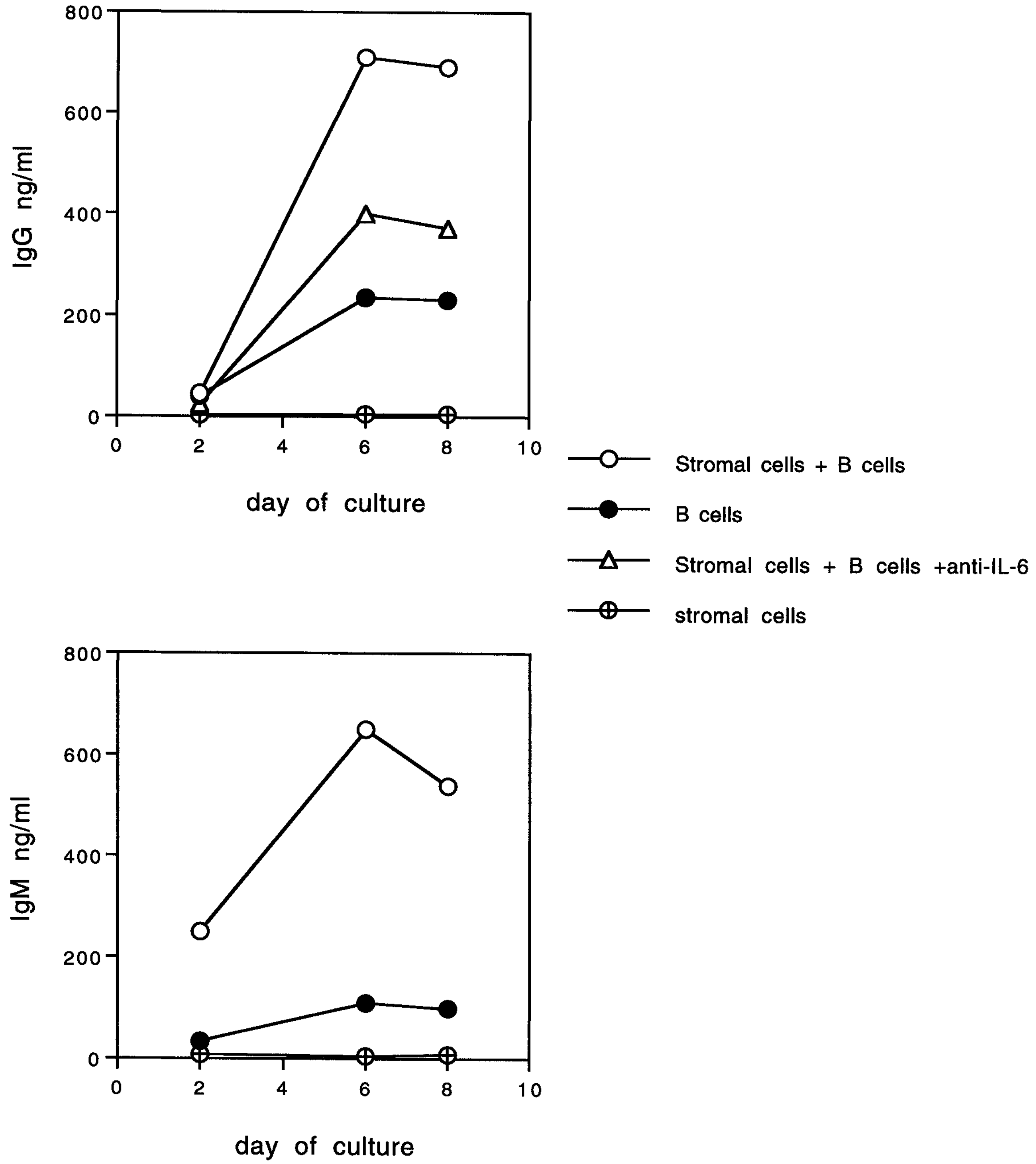

FIGURE 5 Stromal cell lines enhance IgG (A) and IgM (B) production in high-density tonsilar B lymphocytes. The cells were cocultured, and on the days indicated, culture supernatant was harvested and assayed for immunoglobulin concentration. Anti-IL-6 neutralizing antibodies were added at the beginning of culture period at 5 $\mu$g/ml. The figure shows one of five experiments that gave similar results. Stromal cells produced less than 10 ng/ml of IgG and IgM.

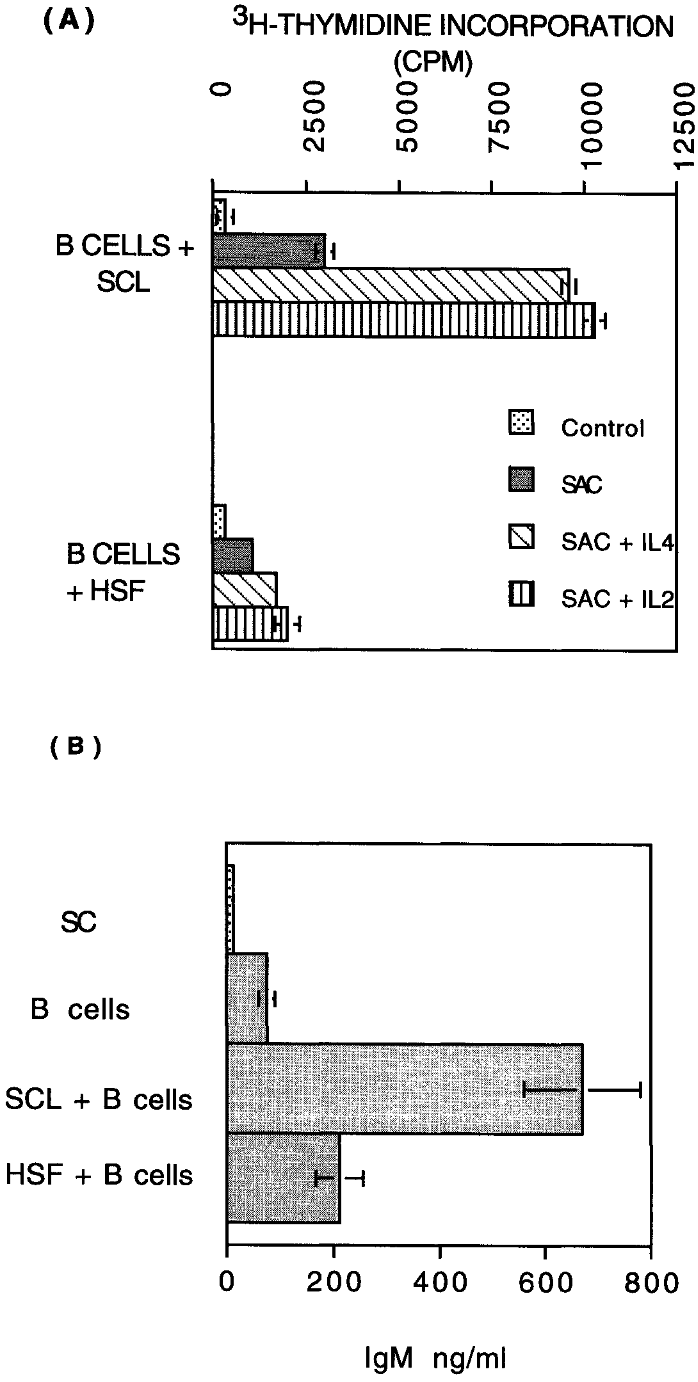

FIGURE 6 Comparison of B-cell stimulating activities of human skin fibroblasts with SCL. (A) B-cell proliferation; irradiated stromal cells and human skin fibroblasts cells were seeded into 96-well plates and incubated overnight. Isolated tonsil B cells were then added together with SAC, IL-2 (100 U/ml), or IL-4 (50 U/ml). Cultures were pulsed with $^3$H-thymidine and processed as described in Materials and Methods. Data presented are mean values from six experiments and SD was less than 10% of the mean for all values. (B) Enhancement of IgM secretion; stromal cells and human skin fibroblasts were cocultured with high-density B lymphocytes for 6 days. Culture supernatants were collected and assayed for IgM concentration by ELISA. Data presented are mean values ±SD of four experiments.

Although cross-linking of surface immunoglobulins can activate B cells to enter the cell cycle, this signal alone may not be sufficient to induce B cells to proliferate. Additional signals from T cells and/or FDC are likely to be required to induce B-cell proliferation. Indeed, studies reported in this paper show that FDC-like cell lines and SAC can promote B-cell proliferation. To obtain maximal proliferation, exogenous IL-4 or IL-2 was needed as well as physical contact since using supernatant from stromal cell lines could not support proliferation to the same extent.

Experiments performed with control fibroblast cell lines showed that they, too, could increase proliferation and IgM production by B cells but to a lower level. Fibroblasts and SCL described here do express some common characteristics. Apart from similar morphology, they both constitutively produce IL-6. Both cells respond to IFN-$\gamma$ and TNF-$\alpha$ with upregulation of ICAM-1. However, unlike fibroblasts, our cells do not produce IL-1. Nevertheless, it is possible that FDC and fibroblasts are related and have some overlapping functions, a possibility already raised by Clark et al. (1995).

Cell lines expressing FDC-like properties have been already reported by other groups. Tsunoda et al. (1990) isolated an FDC cell lihe that, however, does not divide in culture. Lindhout et al. (1994) reported EBV transformed cell lines that had doubling close to 1 week, largely restricting experiments that can be performed with such lines. Other groups (Lindhout et al., 1994; Clark et al., 1995; Kim et al., 1995) reported cells showing similar properties to our SCL growing *in vitro* without exogenous growth factors. All these lines share some common properties. They all originated from human tonsils and are very large, with a fibroblastlike morphology. They all initially expressed markers typical for FDCs, which have been eventually lost after a few days of culture. Our cell types most closely resemble those described by Clark et al. (1995) and by Kim et al. (1995), although they clearly differ in some details. For example, the HK line described by Kim et al. (1995) similarly to our lines did not express VCAM or CD14 and do stimulate B-cell proliferation. Unlike our lines and the FDC-1 cell line (Clark et al., 1995), HK do not stimulate Ig production by B cells. Similarities and differences between different FDC-like cell lines may reflect heterogeneity of FDC *in vivo*.

In conclusion, the results presented here provide further evidence of the role of integrated cell events on the proliferation and function of B cells. Our current studies are aimed at determining the molecular basis of SCL- and B-cell interactions, their possible relevance to B-cell survival, and the effects of such interactions on SCL themselves.

### MATERIALS AND METHODS

### Preparation of Stromal Cells

Tonsils were obtained from six children (age 2-12 years) undergoing routine tonsilectomy. Tonsillar parenchyma was separated from the fibrotic capsule and connective tissue. Tissue was then cut into uniform slices 8 mm in diameter and 300-400-$\mu$m thickness using a Krumdieck tissue slicer (Krumdieck et al., 1980). The slices were incubated in RPMI containing 2% FBS (fetal bovine serum) at 25°C for 6-8 days with continous rocking. The medium was changed every second day. After the incubation period, the remaining tissue was digested (0.25% trypsin, 0.02% EDTA) and depleted of residual CD45$^+$ cells with magnetic beads (mini MACS). The final cell population consisted of large adherent cells with attached lymphocytes. Non-adherent cells were removed by extensive washing with medium and adherent cells and were replenished with fresh medium every 3 days. Adherent cells were trypsinized when they reached confluence. After 2-3 weeks, the growing cells were morphologically uniform, large, and nonphagocytic. Such cells grew in culture for up to 1 month.

Human skin fibroblast cell line (142 BR) was obtained from the ECACC Collection (Salisbury, Wiltshire, England) and was grown in RPMI containing 10% FBS.

### Purification of CD45-Negative Cell Population Using Magnetic Beads

A commercially available system was used (Miltenyi Biotec Ltd. Camberley, Surrey, UK). The cells were first incubated with anti-CD45 mouse monoclonal antibody. After washing with PBS containing 0.5% BSA and 2.5 mM EDTA, the cells were incubated with goat anti-mouse IgG antibodies coupled to magnetic beads according to the manufacturer's instruction. A mixture of labeled and unlabeled cells was passed over a high-gradient magnetic separation column placed in a strong magnetic field. The magnetically stained cells are retained on the column, whereas unstained cells pass through. Cells that passed through the column were used in experiments and contained less then 2% CD45-positive cells as revealed by flow cytometry.

### Immunophenotyping

Stromal cells were assesed for the expression of surface markers using a standard indirect staining method. Briefly, cells were collected after trypsynization (0.2%), washed, resuspended in complete culture medium, and placed in 25-ml tubes on a rocker platform at 37°C overnight, to allow them to reexpress surface markers destroyed by trypsinization. After extensive washing, the cells (about $2 \times 10^5$) were incubated with the different monoclonal antibodies listed in Table I for 30 min at 4°C. Each antibody was diluted to its optimal working concentration in 1% FBS-PBS. As negative controls, cells were incubated with isotype-matched mouse IgG (Dakopats, High Wycombe, England). After two washes in PBS containing 1% FBS and 0.02% sodium azide, the cells were stained with goat anti-mouse IgG $F(ab)_2$ fragments conjugated with FITC (Sigma, Poole, England) at 4°C for 30 min. After the final wash, cells were fixed in 1% paraformaldehyde and analyzed on EPICS XL (Coulter).

### Immunoglobulin Concentration in Culture Supernatants

Ninety-six-well plates (Immulon 1, Dynatech, Billingshurst, England) were coated overnight at 4°C with rabbit anti-human IgA,G,M antiserum (Dakopats), diluted 1/1000 in 0.1 M carbonate buffer, pH 9.6. The plates were blocked for 1 hr at room temperature with PBS 1% BSA. Samples and standards that had been appropriately diluted in PBS/0.5%, BSA/0.1%, Tween 20 were added and incubated for a further 2 hr at room temperature. Isotype-specific, HRP-conjugated, secondary antibodies were added in saturating concentrations and the plates were incubated for an additional 2 hr at room temperature. Ortho-phenyldiamine (OPD) at 0.4 mg/ml in citrate buffer, pH 5.2, containing 0.06% hydrogen peroxide was added as a substrate and the plates were incubated in the dark at room temperature before the reaction was stopped by addition of 2 N $H_2SO_4$. The ODs at 490 nm were measured with an automatic plate reader (Dynastar, Dynatech). All samples were assayed in duplicate.

### Cytokine Production

The cytokine content of stromal cell supernatants was determined using commercially available ELISA kits (R&D Systems, Abingdon, England; Central Laboratory of the Netherlands, Red Cross Blood Transfusion Service, Amsterdam).

### Isolation of B Lymphocytes from Tonsils

Tonsillar mononuclear-cell suspensions were rosetted twice with 2-aminoethylisothiouronium bromide (AET, Sigma)-treated sheep red blood cells. The E-rosette-negative cells obtained were separated into different density fractions on discontinuous Percoll (Sigma) gradients of 55, 45, 40, and 35%. The dense cells that pelleted > 55% Percoll were used for all assays and were > 98% CD20-positive.

### Proliferation Assays

Irradiated stromal cells (2000 rad) were seeded at $2.5 \times 10^3$ cells/well in 96-well flat-bottom microtiter plates and cultured overnight. Purified tonsil B cells were then added to the wells at $2.5 \times 10^5$ cells/well together with formalin-fixed Staphylococcus aureus particles (final dilution $1{:}10^5$) (Sigma). The cells were cocultured for 72 hr. During the last 18 hours of the culture period, the cells were pulsed with $^3$H-thymidine (Amersham Life Sciences, Amersham, England) and then harvested onto the glass fiber filters. $^3$H-thymidine incorporation was measured by liquid scintillation counting using a Packard beta counter. Stromal cells were irradiated in order to reduce background $^{3}H$-thymidine incorporation.

### *Acknowledgements*

This work was funded by EC project on "In Vitro Immunization of Human B Lymphocytes." Martine Deckers was an Erasmus exchange student from the University of Leiden. We thank operating theater staff of the Royal Hospital for Sick Children, Edinburgh, for providing tonsil samples. We also thank Ian Milne and James Black for technical assistance.

### *References*

Banchereau J. and Rousset F. (1992). Human B lymphocytes: Phenotype, proliferation and differentiation. Adv. Immunol. **52**:125.

Clark E.A., Grabstein K.H., Gown A.M., Skelly M., Kaisho T., Hirano T. and Shu G.L. (1995). Activation of B lymphocyte maturation by a human follicular dendritic cell line, FDC-1. J. Immunol. **155**:545.

Gady A., Verma S., Barcena A. and Spits H. (1993). Precursors of CD3+CD4+CD8+ cells in the human thymus are defined by expression of CD34. Delineation of early events in human thymic development. J. Exp. Med. **178**:391.

Jenkinson E.J., Anderson G. and Owen J.J. (1992). Studies on T cell maturation on defined thymic stromal cell population in vitro. J. Exp. Med. **176**:845.

Kim S-H., Zhang X., Klyushnekova E. and Choi Y.S. (1995). Stimulation of germinal center B lymphocyte proliferation by an FDC-like cell line, HK. J. Immunol. **155**:1101.

Kosco M.H. and Gray D. (1992). Signals involved in germinal center reactions. Immunol. Rev. **126**:63.

Kroese F.G.M., Timens W. and Nieuvenhuis P. (1990). Germinal center reaction and B lymphocytes: Morphology and function. Curr. Top. Pathol. **84**:103.

Kroese F.G.M., Wubbena A.S. and Nieuwenhuis P. (1986). Germinal center formation and follicular antigen trapping in the spleen of lethally X-irradiated and recenstituted rats. Immunology **57**:99.

Krumdieck C., Dos Santos J.E. and Ho K.-J. (1980). A new instrument for the rapid preparation of tissue slices. Anal. Biochem. **104**:118.

Lindhout E., Lakeman A., Mevissen M.L.C.M. and de Groot C. (1994). Functionally active Epstein-Barr virus-transformed follicular dendritic cell-like lines. J. Exp. Med. **179**:1173.

MacLennan I.C. (1994). Germinal centres. Annu. Rev. Immunol. **12**:117.

Möller G. (1992). Germinal centers in the immune response. Immunol. Rev. **126**:5.

Tsunoda R., Nakayama M., Onozaki K., Heinen E., Cormann N., Kinet-Donoel C. and Kojima M. (1990). Isolation and long term cultivation of human tonsil follicular dendritic cells. Virchows Arch. B. Cell. Pathol. **59**:95.

